# A Novel and Cost-Effective Do-It-Yourself Handle for Interproximal Stripping: Design, Fabrication, and Clinical Implications

**DOI:** 10.7759/cureus.78998

**Published:** 2025-02-14

**Authors:** Aameer Parkar, Chetan Patil, Pradeep Kawale, Priyanka Khade, Priyanka Razdan

**Affiliations:** 1 Department of Orthodontics and Dentofacial Orthopaedics, Yogita Dental College and Hospital, Khed, IND; 2 Department of Paediatric and Preventive Dentistry, Yogita Dental College and Hospital, Khed, IND

**Keywords:** cost effectiveness, orthodontics, proximal, stainless steel, stripping

## Abstract

Interproximal stripping (IPS), also known as enamel reduction or slenderizing, is a widely used orthodontic technique for resolving mild-to-moderate dental crowding without the need for extractions. Among the various tools available, such as air rotor stripping (ARS), burs, and discs, metallic abrasive strips are commonly used because of their safety and efficiency. However, maneuverability challenges, particularly in the posterior region, and uneven pressure distribution during stripping necessitate the development of improved handling tools. With the surge in the use of clear aligners, IPS has become an important method of gaining space.

This study introduces a simple, economical, and effective do-it-yourself (DIY) handle for IPS, designed to improve the grip, control, and accessibility of abrasive strips. The handle is fabricated using a 19-gauge stainless-steel wire, a universal plier, and a wire cutter. A step-by-step fabrication process ensures the construction of a stable ergonomic instrument that securely engages the IPS strip, providing enhanced control in both the anterior and posterior regions. The handle design minimizes operator fatigue, reduces the risk of strip slippage, and allows for precise enamel reduction.

Compared to conventional IPS tools, such as oscillating strips and ARS, the DIY handle offers significant advantages. It reduces mechanical vibrations and heat generation, thereby minimizing the potential damage to the dental pulp. Additionally, it is reusable, cost-effective, and easy to sterilize, making it a sustainable option in orthodontic practice. The compatibility of the handle with both single- and double-sided abrasive strips further enhances its versatility.

This innovative tool is an accessible solution for clinicians, particularly in resource-limited settings. By addressing the limitations associated with existing IPS methods, the DIY handle improves the precision and safety of enamel reduction procedures while optimizing patient comfort and treatment outcomes.

## Introduction

Interproximal stripping (IPS), which is also referred to as enamel reduction or slenderizing, is a technique frequently used in the field of orthodontics to rectify crowding and irregularities in dental alignment. The procedure entails the systematic removal of a diminutive quantity of enamel from the proximal surfaces of the teeth, particularly within the interproximal regions that exist between adjacent dentitions. The objective of this intervention is to generate additional space within the dental arch, thereby facilitating the optimal alignment of teeth and ultimately fostering harmonious and functional occlusion. The increasing prevalence of interproximal stripping can be attributed to its minimally invasive characteristics and efficacy in addressing mild to moderate crowding concerns, which frequently serve as a viable alternative to the extraction of teeth [1,2].

Various instruments used for IPS include stainless steel strips, manual disk hand tools, high-torque diamond disks, air rotor stripping (ARS) burs, and diamond disks [3]. The use of a metallic abrasive strip is regarded as the most secure and widely used method among the aforementioned techniques. The strip may be positioned in the anterior region with relative ease; however, its utilization in the posterior region presents challenges because of the difficulty associated with maintaining a grip on the strip with the fingers while performing the stripping procedure on the posterior dentition [4]. The application of rotary cutting instruments has the potential to adversely affect dental pulp owing to exposure to mechanical vibrations and heat generation. Furthermore, the substantial diameter of the disk impedes the visibility of the operative field. Additionally, the phenomenon of fracturing a segment is a prevalent issue associated with the use of disks [5]. To avoid this inconvenience, a simple and economical method of fabricating a strip holder from routine laboratory materials is presented in this article.

## Technical report

**Figure 1:**
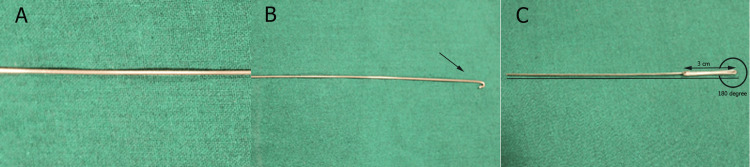
(A) Straight stainless-steel wire; (B) Fabrication of the hook; (C) First bend of 180 degrees

The construction of the do-it-yourself (DIY) handle for IPS follows a structured wire-bending process that ensures durability, ergonomic design, and optimal functionality. The process begins by selecting a 10-inch length 19-gauge stainless steel wire, which serves as the base material for the handle (Figure 1A). A curved hook is formed at one end of the wire using a universal plier (Figure 1B). This hook is essential because it acts as an anchorage point for the interproximal enamel reduction (IPR) strip, which has holes, preventing it from slipping during the stripping procedure. From this hook, 3 cm is measured, and a horizontal bend (first bend) of 180 degrees is made (Figure 1C). This bend forms the initial framework of the handle, providing stability and structure for the overall design.

**Figure 2:**
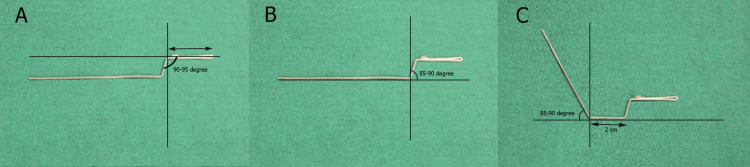
(A) Second bend of 90-95 degrees 3 cm from the hook; (B) Third bend of 85-90 degrees; (C) Fourth bend of 85-90 degrees is positioned 2 cm away from the second bend

Following this, at 3 cm from the first bend of 90-95 degrees is introduced (Figure 2A). This bend helps to align the handle for better ergonomic control during the IPS. Moving 2 cm further, a third bend of 85-90 degrees is placed (Figure 2B) to ensure that the wire maintains a proper functional grip. Subsequently, a fourth bend of 85-90 degrees is positioned 2 cm from the third bend (Figure 2C). These sequential bends are essential for creating an angled structure that allows better maneuverability in the interdental space while ensuring minimal operator fatigue.

**Figure 3:**
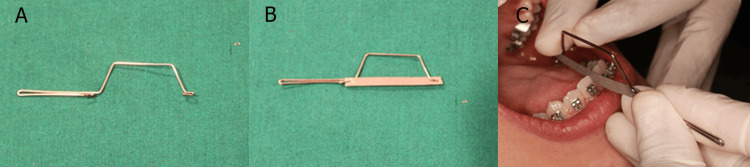
(A) Fabricated do-it-yourself (DIY) handle with hooks; (B) Engaging the interproximal strip to the hooks; (C) Using the DIY handle for interproximal stripping

After the bending is completed, a second hook is formed at the opposite end of the wire (Figure 3A). This hook mirrors the first hook and allows the IPS strip to be held in place securely, ensuring stability during use. Once the wire structure is finalized, it is checked for symmetry, smoothness, and proper angulation to ensure that it does not cause discomfort to the patient or difficulty in handling (Figure 3B). This handle can be used with single- or double-sided abrasive strips in the interdental area for the IPS (Figure 3C).

Several precautionary measures should be taken during the fabrication process to ensure the safety, efficiency, and durability of the DIY handle. First, a stainless-steel wire with an appropriate gauge (19-gauge) must be used, as thinner wires may lack the necessary strength, while thicker wires can become difficult to manipulate. Sharp edges or burs formed after cutting the wire should be smoothed using a file to prevent injury to the patient or operator. Precise measurement of each bend is essential to maintain the balance and stability of the handle because incorrect angulations can reduce its effectiveness. Additionally, hooks at both ends should be tightly formed to hold the IPR strip securely, preventing any loosening or slippage during the stripping procedures. When using a DIY handle, adequate infection control measures should be observed, including sterilization before each use to prevent cross-contamination. The handle should be checked for any signs of bending, deformation, or weakening before each procedure, to ensure continued effectiveness. During IPS, the operator should apply controlled force to avoid excessive enamel reduction and ensure patient comfort.

## Discussion

Interproximal stripping, or enamel reduction, has gained significant traction in orthodontic practice as a minimally invasive and effective method for addressing mild-to-moderate dental crowding and irregularities. This technique not only avoids the need for extraction in many cases but also allows for enhanced control over tooth alignment, enabling more harmonious and functional occlusion [5]. Among the various instruments available for IPS, the use of metallic abrasive strips has been widely favored owing to its safety, simplicity, and efficiency. However, challenges in accessing the posterior region of the dental arch and irregular cutting of the enamel surface due to uneven pressure applied by the hands during stripping procedures [1,6] have necessitated the development of innovative tools such as the DIY handle, as described in this article.

The DIY handle for IPS is a practical and economical solution that addresses several limitations associated with traditional methods. Fabricated using readily available materials, including a 19-gauge stainless steel wire, universal plier, and wire cutter, the handle offers a versatile and ergonomic design for securing abrasive strips. The detailed step-by-step fabrication process ensures precision and ease of use, enabling clinicians to construct the tool chairside in a minimal time. One of the key advantages of the DIY handle is its compatibility with both single- and double-sided abrasive strips, which provides flexibility in its application.

From a clinical perspective, the ergonomic design of the DIY handle enhances accessibility and maneuverability when abrasive strips are used in both the anterior and posterior regions. Unlike traditional handheld strips, which often require significant dexterity and can lead to operator fatigue, the handle offers improved grip and control [7]. This feature is particularly beneficial when working in the posterior region, where maintaining a stable hold on the strip is challenging. In addition, the reduced risk of strip slippage or misplacement contributes to enhanced safety and precision during the procedure [8].

Another notable advantage of the DIY handle is its reusability. The handle fabricated from stainless steel can be sterilized and reused, making it a cost-effective and environmentally sustainable option for clinicians. The minimal armamentarium required for its construction further enhances practicality, particularly in resource-limited settings. The absence of stress points in the wire during fabrication ensures the structural integrity of the handle, thereby reducing the likelihood of deformation or breakage during use.

The DIY handle also addresses common issues associated with other IPS tools, such as oscillating strips [9]. For instance, oscillating instruments often generate mechanical vibrations and heat, posing a risk to dental pulp and patient comfort [10]. Banga et al. [10] observed a minimal rise in pulpal temperature with the use of manual abrasive strips, compared to oscillating strips, ARS, or burs. Sehgal et al. [11] investigated the variations in pupal temperature employing diverse IPS methodologies and discovered that the utilization of diamond burs in a micromotor handpiece devoid of coolant led to the most significant elevation in temperature (3.5°C), succeeded by handheld diamond strips (2.8°C). Gul Amuk et al. [12] documented that the use of a tungsten bur in conjunction with a perforated disk while employing air cooling, resulted in the most significant elevation in pulpal temperature. In contrast, the DIY handle offers a controlled and precise stripping process that minimizes the risk of complications while maintaining patient comfort.

## Conclusions

In summary, the DIY handle epitomizes a novel user-centric instrument for IPS. Its merits, encompassing straightforward insertion and removal, fabrication from readily available materials, ease of fabrication, reduced wastage of abrasive strips, and improved accessibility, render it an essential component of the orthodontist’s toolkit. By overcoming the shortcomings of conventional IPS methodologies, the DIY handle not only enhances clinical efficacy but also improves the overall experience for both patients and practitioners. Its cost-effectiveness, straightforward design, and practicality underscore the potential for its widespread adoption in orthodontic practice.
